# Characterization and phylogenetic analysis of the complete mitochondrial genome of *Fomitopsis palustris* (Berk. & M.A. Curtis) Gilb. & Ryvarden, 1985 (Polyporales: Fomitopsidaceae)

**DOI:** 10.1080/23802359.2025.2509806

**Published:** 2025-05-25

**Authors:** Wei Gao, Shuyi Chen, Qiang Li

**Affiliations:** a Clinical Medical College & Affiliated Hospital of Chengdu University, Chengdu University, Chengdu, China; b School of Food and Biological Engineering, Chengdu University, Chengdu, China

**Keywords:** Mitochondrial genome, fungi, evolution, phylogeny

## Abstract

*Fomitopsis palustris* (Berk. & M.A. Curtis) Gilb. & Ryvarden, 1985 is a well-known brown-rot basidiomycete. However, the complete mitochondrial genome of *F. palustris* from China remains uncharacterized. In this study, we present the complete mitochondrial genome sequence of *F. palustris*, which spans 64,223 bp and has a GC content of 23.89%. The genome contains 15 essential protein-coding genes, 10 other ORFs, nine intronic ORFs, 26 tRNAs, and two rRNAs. Phylogenetic analysis via Bayesian inference (BI) was conducted to elucidate the evolutionary relationships among 10 closely related species, revealing well-supported clades and revealing the close relationship between *F. palustris* and *F. pinicola*.

## 1. Introduction

*Fomitopsis palustris* (Berk. & M.A. Curtis) Gilb. & Ryvarden, 1985, a brown-rot basidiomycete belonging to the Fomitopsidaceae family, is widely distributed in temperate and boreal forests (Nurul-Aliyaa et al. 2023; Tanaka et al. 2023). This species has garnered considerable attention because of its ecological importance as a major wood decomposer and its potential biotechnological applications (Yoon and Kim 2005; Zhao et al. 2018). *F. palustris* is known to produce a variety of secondary metabolites, including polysaccharides, terpenoids, and phenolic compounds, which exhibit antioxidant, anti-inflammatory, and antitumor activities (Song et al. 2008; Hong et al. 2017; Tanaka et al. 2020; Liang et al. 2024).

In recent years, there has been an increasing focus on the mitochondrial genome of higher fungi because of its significant involvement in energy production, cellular respiration, and metabolic pathways (Osiewacz 2002; Chatre and Ricchetti 2014; MacKillop and Kennell 2022; Gao et al. 2024). Furthermore, the mitochondrial genome has been identified as a promising tool for exploring fungal phylogeny (Li, Bao, et al. 2022; Li, Li, et al. 2022). Nevertheless, the assembly and analysis of the mitochondrial genome in higher fungi pose challenges because of its intricate structure and the existence of repetitive sequences (Li, Chen, et al. 2018; Li et al. 2023). Currently, the understanding of the characteristics of the mitochondrial genome within the *Fomitopsis* genus is limited, with only two complete mitochondrial genomes documented thus far (Österman-Udd and Lundell 2023). The mitochondrial genome of *F. palustris* from Japan has been reported (Tanaka et al. 2017). However, mitochondrial genome varies greatly among fungi from different places. So far, no complete mitochondrial genome of *F. palustris* from China has been reported (Österman-Udd and Lundell 2023).

The current study conducts a sequencing, assembly and detail analysis of the complete mitochondrial genome of *F. palustris* from China. Our discoveries enrich the current understanding of fungal mitochondrial genome variations.

## 2. Materials and methods

### 2.1. Sample collection

**Figure 1.**
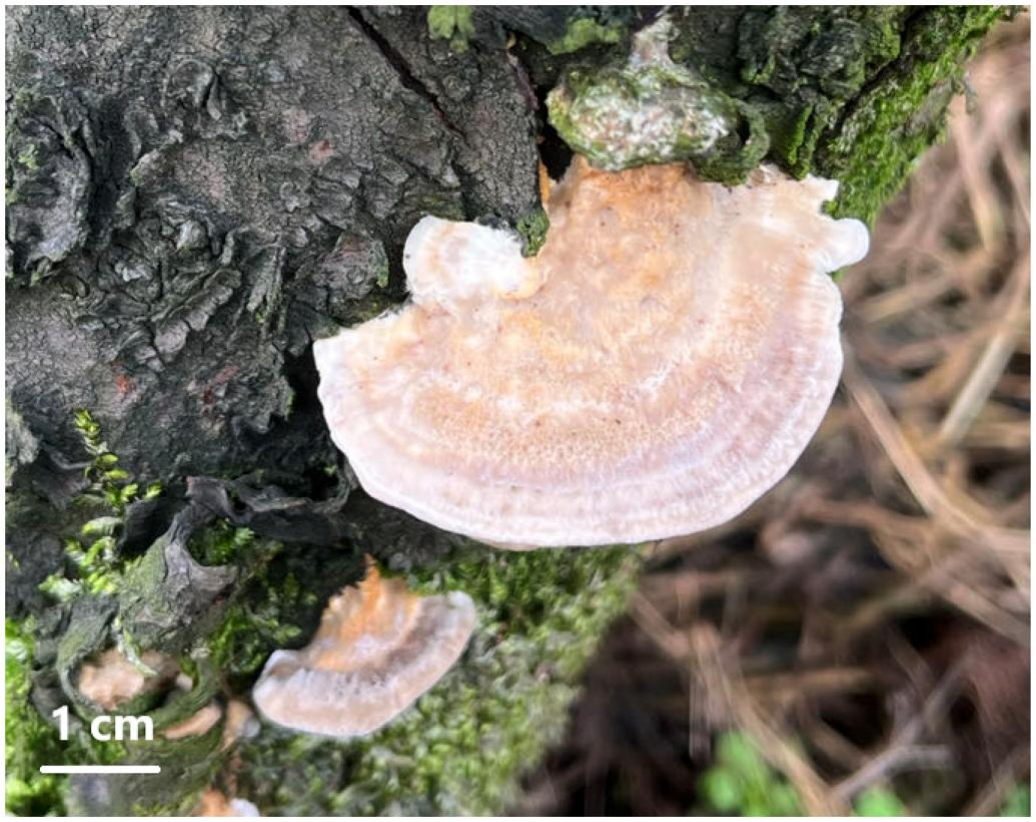
Morphology of *Fomitopsis palustris* fruiting bodies. A photo of the species was taken by Wei Gao using a camera (Canon EOS 5D Mark IV, Canon Inc., Ōta, Japan).

A sample of *F. palustris* was obtained from a peach tree located in Chengdu, Sichuan, China (30.56N, 104.18E), in 2024 (Figure 1). The identification of the samples relied on morphological examination and the use of nuclear genomic molecular markers, specifically the ITS, translation elongation factor 1-α (tef1α) gene, and partial nuclear ribosomal large subunit (nrLSU), as detailed in previous research (Liu et al. 2021, 2022; Spirin et al. 2024). A specimen was archived at the Culture Collection Center of Chengdu University with voucher number Fpa02 (for further inquiries, contact Wei Gao: gaowei@cdu.edu.cn).

### 2.2. Mitochondrial genome assembly and annotation

The mitochondrial genome of *F. palustris* was assembled and annotated following established protocols (Li, Yang, et al. 2020; Li et al. 2021). Fungal DNA was extracted from fruiting bodies via a fungal DNA extraction kit (Norcross, GA). The NEBNext^®^ Ultra™ II DNA Library Prep Kit (NEB, Beijing, China) was subsequently utilized for sequencing library preparation according to the manufacturer’s instructions. Sequencing was performed on the Illumina HiSeq 2500 platform (Illumina, San Diego, CA), and a total of 13,929,948 paired-end raw reads (approximately 2.09 Gbp raw bases) were obtained. The quality control measures included the removal of low-quality sequences via ngsShoRT (Chen et al. 2014) and the elimination of adapter reads via AdapterRemoval v2 (Schubert et al. 2016). The mitochondrial genome of *F. palustris* was assembled *de novo* via NOVOPlasty version 4.3.3, with a k-mer size of 29 (Dierckxsens et al. 2017). The coverage depth map reveals an average depth of 3860.66×, as illustrated in Figure S1. Annotation of the mitochondrial genome followed established protocols, employing the MFannot tool (Valach et al. 2014; Lang et al. 2023) and MITOS2 (Bernt et al. 2013). Protein-coding genes (PCGs) and open reading frames (ORFs) were annotated or validated through BLASTP searches against the NCBI nonredundant protein sequence database (Bleasby and Wootton 1990). We identified tRNA genes in the mitochondrial genome of *F. palustris* using tRNAscan-SE v1.3.1 (Lowe and Chan 2016). Gene structures containing introns and the overall mitochondrial genome were visualized and graphically represented via the PMGmap online web tool (Zhang et al. 2024).

### 2.3. Phylogenetic analysis

The phylogenetic tree was constructed via established methods (Li, Zhang, et al. 2022), with individual mitochondrial genes aligned (excluding introns) via MAFFT v7.037 software (Katoh et al. 2019). The aligned mitochondrial genes were then merged via SequenceMatrix v1.7.8 to create a unified mitochondrial dataset (Vaidya et al. 2011). An initial partition homogeneity test was conducted with PAUP v 4.0b10 (Swofford 2002) to identify potential phylogenetic discrepancies among different mitochondrial genes, following literature (Xiang et al. 2013). The optimal partitioning schemes and evolutionary models for the combined mitochondrial dataset were determined via PartitionFinder 2.1.1 (Lanfear et al. 2017). Phylogenetic trees were constructed through Bayesian inference (BI) with MrBayes v3.2.6 software (Ronquist et al. 2012). The Bayesian inference analysis involved two independent runs with four chains (three heated and one cold) each, running simultaneously for 2 × 10^6^ generations. Sampling was performed every 1000 generations, discarding the first 25% of the samples as burn-in. Bayesian posterior probabilities (BPPs) were calculated via the remaining trees to generate a 50% majority rule consensus tree. We also construct the phylogenetic tree using maximum-likelihood (ML) method based on the combined gene set using RAxML v8.0.0 (Stamatakis 2014).

## 3. Results

**Figure 2.**
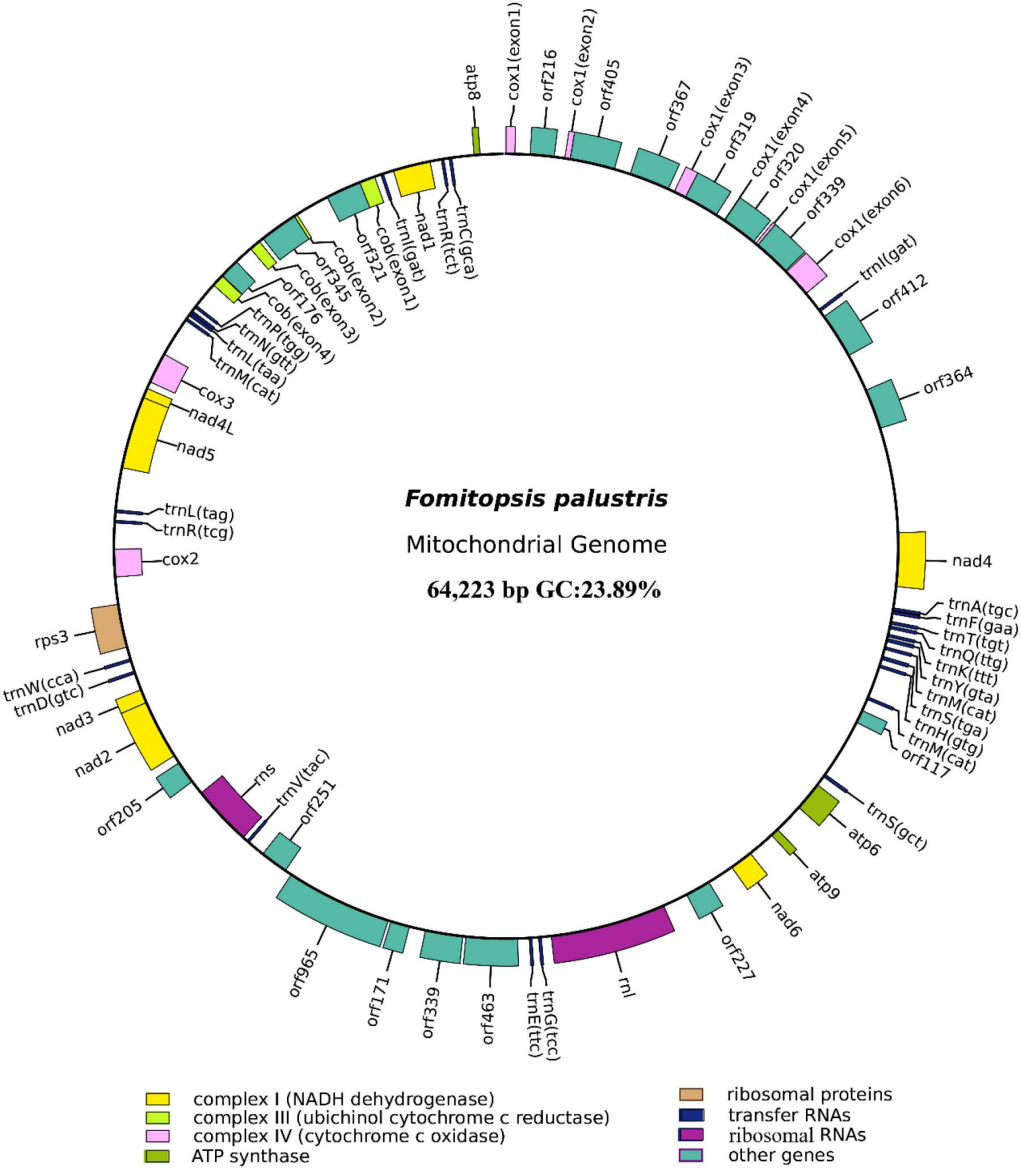
Circular mitochondrial genome map of *Fomitopsis palustris*. Different color blocks represent different genes. Genes located in the outer loop indicate that they are on the direct strand, whereas genes located inside the loop indicate that they are on the reverse strand.

**Figure 3.**
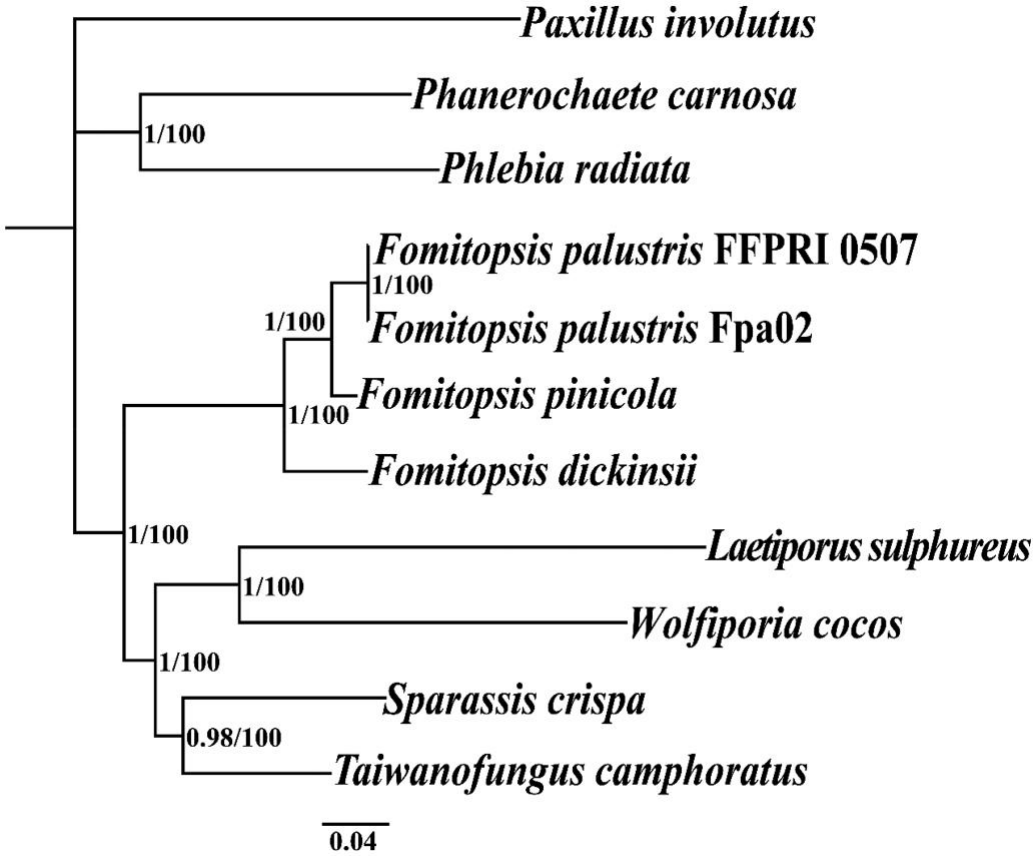
Phylogenetic tree generated from 14 concatenated mitochondrial PCGs (*atp6*, *atp8*, *atp9*, *cob*, *cox1*, *cox2*, *cox3*, *nad1*, *nad2*, *nad3*, *nad4*, *nad4L*, *nad5*, and *nad6*) from *Fomitopsis palustris* and closely related genera, including *Phanerochaete*, *Phlebia*, *Laetiporus*, *Wolfiporia*, *Taiwanofungus*, and *Sparassis,* based on Bayesian inference (BI) and maximum-likelihood (ML) method. *Paxillus involutus* (MK993563) was used as the outgroup (Li, Ren, et al. 2020). Support values are Bayesian posterior probabilities (BPPs, before slash) and bootstrap values (BS, after slash). The accession numbers of the sequences were as follows: *Fomitopsis palustris* FFPRI 0507 (AP017926) (Tanaka et al. 2017), *Taiwanofungus camphoratus* (MH745717) (Wang, Jia, et al. 2020), *Phanerochaete carnosa* (MT090080) (Wang, Song, et al. 2020), *Fomitopsis palustris* (PQ638955), *Laetiporus sulphureus* (MG519331) (Li, Yang, et al. 2018), *Phlebia radiata* (HE613568) (Salavirta et al. 2014), *Wolfiporia cocos* (MT079861) (Chen et al. 2020), *Sparassis crispa* (MN722635) (Bashir et al. 2020), *Fomitopsis dickinsii* (OR544899), and *Fomitopsis pinicola* (OX579598) (Österman-Udd and Lundell 2023).

The mitochondrial genome of *F. palustris* spans 64,223 bp, with a GC content of 23.89%. In *F. palustris*, the mitochondrial genome comprises 38.24% adenine, 12.04% guanine, 37.87% thymine, and 11.85% cytosine. Analysis of the mitochondrial genome of *F. palustris* revealed 34 ORFs, containing 15 core PCGs (*cox1*, *cox2*, *cox3*, *atp6*, *atp8*, *atp9*, *cob*, *nad1*, *nad2*, *nad3*, *nad4*, *nad4L*, *nad5*, *nad6*, and *rps3*), in addition to 10 free-standing ORFs and nine intronic ORFs, as shown in Figure 2. This genome contains eight introns, consisting of seven group IB and one group ID, with intronic ORFs predominantly encoding LAGLIDADG-homing endonucleases. Notably, two free-standing ORFs encode DNA polymerase. Furthermore, the *F. palustris* mitochondrial genome contains two ribosomal RNA genes (*rns* and *rnl*) and 26 transfer RNA genes. Gene structures containing introns are presented in Figure S2. Phylogenetic analysis revealed the closest evolutionary relationship of *F. palustris* to *F. pinicola* (Österman-Udd and Lundell 2023) based on the GTR + I + G model, as illustrated in Figure 3.

## 4. Discussion and conclusions

The availability of the mitochondrial genome can contribute to a more thorough comprehension of species’ phylogenetic relationships (Zhang et al. 2020, 2022, 2023; Ren et al. 2021). The mitochondrial genomic variation of the same fungus from different regions is very large. In this study, we conducted whole-mitochondrial-genome sequencing of a *Fomitopsis* species and identified a genome length of 64,223 bp with a GC content of 23.89%. The mitochondrial genome of *F. palustris* from China is 744 bp larger and the GC content is 0.13% lower than that of *F. palustris* from Japan (AP017926). In addition, the *F. palustris* from China has one more PCGs and one more intron than the *F. palustris* from Japan, indicating large variation in the *Fomitopsis* species. Phylogenetic analysis via the BI and ML methods revealed a close relationship between *F. palustris* and *F. pinicola* among 10 closely related species, with strong support for major clades (Österman-Udd and Lundell 2023). This research enhances our understanding of the differentiation of *Fomitopsis* species, as well as the evolutionary trends and diversity of the mitochondrial genome in this important fungal genus.

## Supplementary Material

Supplementary figure1.docx

## Data Availability

The genome sequence data that support the findings of this study are openly available in the NCBI GenBank at https://www.ncbi.nlm.nih.gov/ under accession no. PQ638955. The associated BioProject, SRA, and Bio-Sample numbers are PRJNA1190611, SRR31510980, and SAMN45050664, respectively.
