# Supplementary material for: Characterization and phylogenetic analysis of the complete mitochondrial genome of Fomitopsis palustris (Berk. & M.A. Curtis) Gilb. & Ryvarden, 1985 (Polyporales: Fomitopsidaceae)
Source: Mitochondrial DNA B Resour. 2025 May 25;10(6):532–6. doi: 10.1080/23802359.2025.2509806 (PMC12107645; doi:10.1080/23802359.2025.2509806)
Supplement: Supplementary figure1.docx [file TMDN_A_2509806_SM6062.docx]

**Characterization and phylogenetic analysis of the complete mitochondrial genome of** ***Fomitopsis palustris*** **(Berk. & M.A. Curtis) Gilb. & Ryvarden, 1985 (Polyporales: Fomitopsidaceae)**

**Wei Gao^1^, Shuyi Chen^2^, Qiang Li ^2^***

**1 Clinical Medical College & Affiliated Hospital of Chengdu University, Chengdu University, Chengdu, Sichuan, China;**

**2 School of Food and Biological Engineering, Chengdu University, Chengdu, Sichuan, China;**

**ORCID:**

**Wei Gao (0000-0002-0522-7234)**

**Shuyi Chen (0009-0006-8307-5762)**

**Qiang Li (0000-0002-9772-8617)**

*** Correspondence: leeq110@126.com (Q.L.)**

**Phone: +86-15196619794;**

***Present address: 2025# Chengluo Avenue, Longquanyi District, Chengdu City, Sichuan Province, China.**


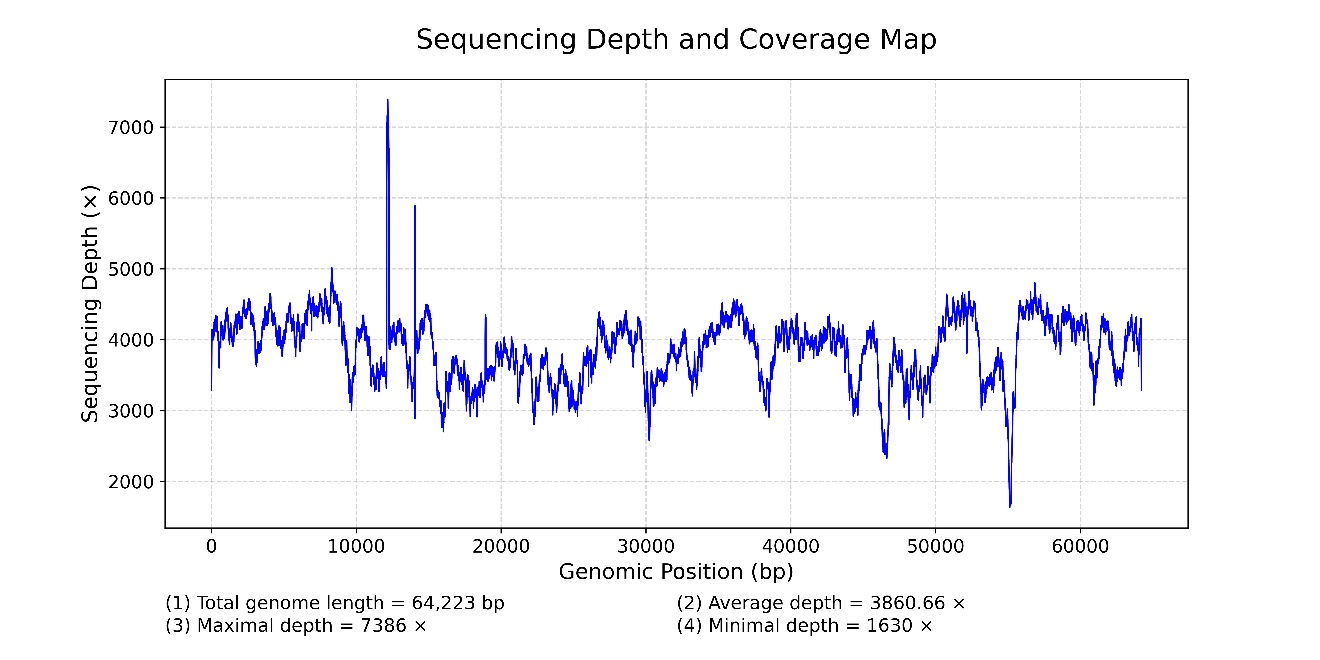


**Figure S1** Sequencing depth and coverage map of *Fomitopsis palustris* mitochondrial genome.


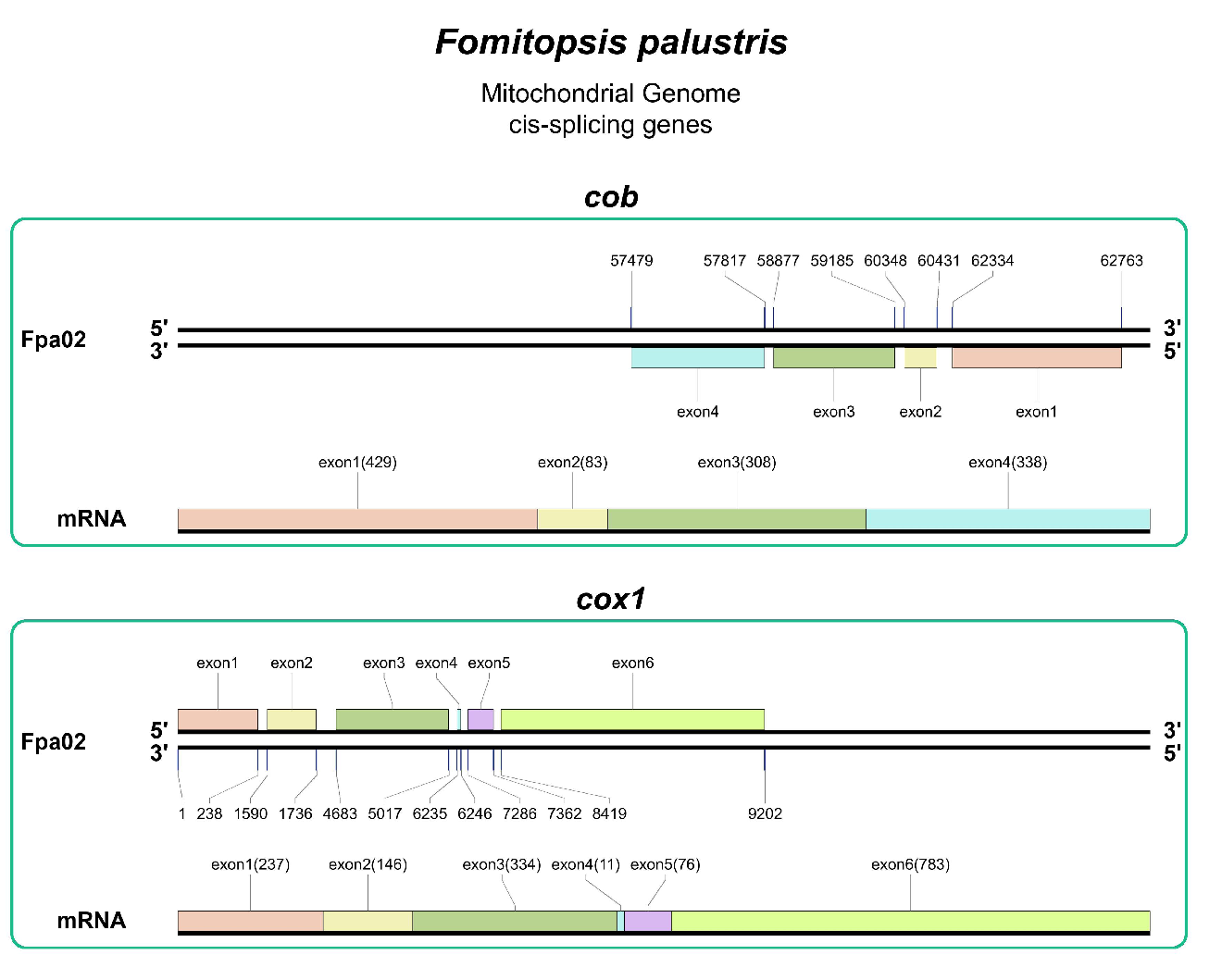


**Supplementary Figure S2** Cis-splicing genes of the *Fomitopsis palustris* mitochondrial genome.
